# Phase Diagram of Continuous Binary Nanoalloys: Size, Shape, and Segregation Effects

**DOI:** 10.1038/srep41990

**Published:** 2017-02-07

**Authors:** Mingjin Cui, Haiming Lu, Haiping Jiang, Zhenhua Cao, Xiangkang Meng

**Affiliations:** 1 National Laboratory of Solid State Microstructures, Collaborative Innovation Center of Advanced Microstructures, College of Engineering and Applied Sciences, Institute of Materials Engineering, Nanjing University, Jiangsu, PR China

## Abstract

The phase diagrams of continuous binary nanoalloys are important in providing guidance for material designs and industrial applications. However, experimental determination of the nano-phase diagram is scarce since calorimetric measurements remain quite challenging at the nanoscale. Based on the size-dependent cohesive energy model, we developed a unified nano-thermodynamic model to investigate the effects of the size, shape, and segregation on the phase diagrams of continuous binary nanoalloys. The liquidus/solidus dropped in temperature, two-phase zone was narrowed, and the degree of surface segregation decreased with decrease in the size or increase in the shape factor. The congruent melting point of Cu-Au nanoalloys with and without segregation is linearly shifted to higher Au component and lower temperature with decreasing size or increasing shape factor. By reviewing surface segregated element of different binary nanoalloys, two segregation rules based on the solid surface energy and atomic size have been identified. Moreover, the established model can be employed to describe other physicochemical properties of nanoalloys, e.g. the cohesive energy, catalytic activation energy, and order-disorder transition temperature, and the validity is supported by available other theoretical prediction, experimental data and molecular dynamic simulations results. This will help the experimentalists by guiding them in their attempts to design bimetallic nanocrystals with the desired properties.

Continuous binary nanoalloys have attracted considerable interests owing to their unique physicochemical properties^1^,^2^,^3^,^4^,^5^,^6^,^7^,^8^,^9^,^10^,^11^,^12^,^13^,^14^,^15^,^16^,^17^,^18^,^19^,^20^,^21^,^22^,^23^,^24^,^25^. For example, Ni-Cu nanoalloys are recognized as efficient catalysts and promising materials for electromechanical devices^3^,^4^,^7^. To fully understand the behavior of continuous binary nanoalloys for applications, a knowledge of the phase diagram is required, since the phase diagram can provide important guidance for tuning their thermodynamic and other properties to achieve optimum device performance. However, experimental determination of the nano-phase diagram is scarce since calorimetric measurements remain quite challenging at the nanoscale^1^,^20^,^25^. Consequently, theoretical methods and computer simulations have become the commonly used methods for predicting the nano-phase diagrams in recent years.

The most applicable theoretical method for bulk and nanoscale phase diagram modelling is the semi-empirical CALPHAD method by defining the Gibbs free energy term for all phases, noting that the CALPHAD method is based on classical thermodynamics in which the number of atoms and the volume are large^12^. However, the CALPHAD method is limited to large spherical nanoparticles (approx. >10 nm in diameter) because energy contributions from the crystal vertices, edges, and faces cannot be neglected when the size is below 10 nm^12^. In contrast, the methods of computer simulation, such as Metropolis Monte Carlo (MMC) methods, Molecular Dynamic (MD) simulations and Density Functional Theory (DFT) are limited to systems containing a few hundred or thousand number of atoms^13^,^14^,^15^,^16^,^17^. Nano-thermodynamics served as a bridge between macroscopic and nanoscopic systems, and it is essential to investigate the size-induced variation of physicochemical properties in nanomaterials^18^,^19^. Recently, several nano-thermodynamic works have been done to predict the phase diagrams of nanoalloys over the entire composition range^1^,^20^,^21^,^22^,^23^,^24^,^25^. For example, Liang *et al*. modelled the size dependence of binary continuous phase diagrams of metals, semiconductors, ceramics and organic nanocrystals^22^, however, the effects of shape and segregation were not included. Recently, Guisbiers *et al*. considered the size, shape, and segregation effects on the phase diagrams of Au-Ag, Au-Cu, and Cu-Ni polyhedral nanoparticles and presented two new segregation rules to determine the nature of the segregated element at the surface of bimetallic nanoalloys^1^,^20^,^24^,^25^. However, the results and discussions in the work of Guisbiers are quite questionable^26^, because their employed values of surface energy were calculated by the full charge density (FCD) method with the generalized gradient approximation (GGA). Note that it has been shown that GGA needs to be corrected due to the exclusion of surface electron self-interactions^27^. For example, the surface energies of solid Au, Cu, and Ni (111) were determined to be 1.28, 1.95, and 2.01 J/m^2^ by GGA^27^, which are 15%, −9% and 18% different from the corresponding experimental data of 1.50 (or 1.51), 1.79 (or 1.83), and 2.45 (or 2.38) J/m^2^ and other theoretical results of 1.52, 1.83, and 2.44 J/m^2^ 27. Moreover, in FCD calculations with GGA, there are often exceptions where the most close-packed surface does not have the lowest surface energy values or there is a weak orientation dependence^27^. The authors of reference 1 used the above questionable data, resulting in the prediction of surface Ni segregation in Cu-Ni system, contrary to the fact that Cu segregates to the surface in this system^28^,^29^. As a result, the segregation rules proposed by Guisbiers are self-contradictory and incorrect^26^. Therefore, it is necessary to develop a reasonable model to investigate the effects of size, shape, and segregation on the phase diagrams of continuous binary nanoalloys and propose logical segregation rules to predict the nature of the segregated element.

## Formula

When a binary system is in thermodynamic equilibrium, the chemical potentials of component A (or B) in the solid phase and liquid phase are equal. In this regard, for a regular solution, the solidus and liquidus of continuous binary alloys have been deduced as^22^,


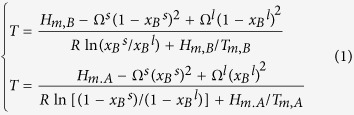


*R* denotes the ideal gas constant and *x* is the composition where the superscripts *s* and *l* denote the solid and liquid phases at given temperature *T. T*_m_, *H*_m_, and Ω are the melting temperature, melting enthalpy, and interaction parameter, respectively. The model has successfully predicted the bulk binary regular solution phase diagrams of metals^1^,^20^,^22^,^25^.

The surface segregation is a critical issue affecting the surface properties and electrocatalysts of binary alloys. The surface segregation of bulk alloys has been well studied experimentally and theoretically^28^,^29^,^30^. According to semi-empirical theories, surface segregation is caused by the difference of surface energy between two components and lattice strain energy arising from lattice mismatch. Thus, Tomanek *et al*. determined the surface composition of bulk solidus (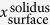) as^28^,


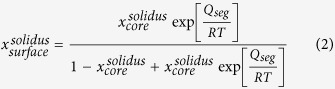


where 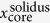 is the bulk solidus composition. The heat of segregation *Q*_seg_ is the work involved in exchanging a surface atom and a bulk atom.

To calculate the phase diagrams at the nanoscale, the size- and shape-dependent thermodynamic parameters are required. On the basis of Lindemann’s criterion of melting, the melting temperature is linear to the force constant of the lattice vibration where the latter can be expressed by the cohesive energy *E*_c_, i.e. *E*_c_ ≈ *m*_1_*T*_m_ with *m*_1_ being a constant^31^. Similar linear relations between the melting enthalpy or the heat of segregation and cohesive energy also exist for metals, namely *E*_c_ ≈ *m*_2_*H*_m_ and *E*_c_ ≈ *m*_3_*Q*_seg_ where *m*_2_ is a constant and *m*_3_ is a material parameter related to the structure^1^,^20^,^25^,^31^. When the nanocrystals have the same structure as the corresponding bulk, *m*_1_, *m*_2_, and *m*_3_ are size-independent, and thus the above three relationship can be extended to the range of nanoscale with the same forms. As a result, *T*_m_, *H*_m_, and *Q*_seg_ have the same size dependence as the cohesive energy^31^, namely


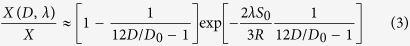


where *X* denotes *E*_c_, *T*_m_, *H*_m_, or *Q*_seg_. *S*_0_ is the bulk solid-vapor transition entropy of crystal. *D*_0_ is a critical size at which all atoms of crystal are located on its surface, which can be determined^31^ as *D*_0_ = 2(3 − *d*)*h* with *d* and *h* being the dimensionality and the nearest atomic distance. *d* = 0, 1, and 2 for nanoparticle, nanowire, and thin film^31^. The shape factor *λ*, describing the shape effect on the ratio of surface atoms to the total atoms, has been determined as^32^,


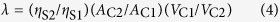


where *η*_S_ denotes surface packing density, *A* and *V* are the surface area and volume of nanoparticles, and the subscripts 1 and 2 denote nanoparticles with a spherical shape and with other shape, respectively. Obviously, the *λ* value for spherical nanoparticle is equal to one according to Eq. (4) and *λ* for other polyhedral particles are calculated and listed in Table 1.

**Figure 1:**
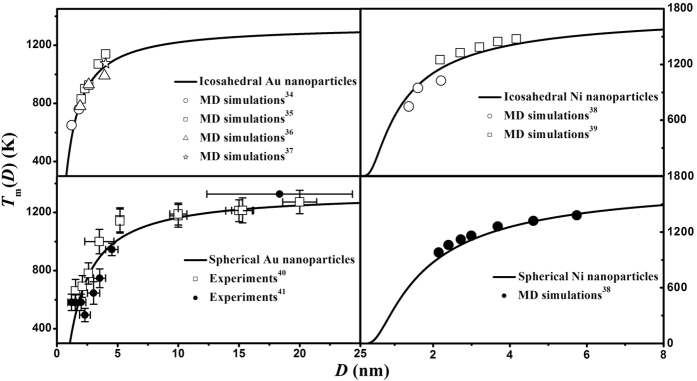
Comparisons of *T*_*m*_(*D,λ*) functions for spherical and icosahedral Au and Ni nanoparticles between the predictions in terms of Eq. (3) and the corresponding MD simulation results^34^,^35^,^36^,^37^,^38^,^39^ or experimental data^40^,^41^.

As shown in Fig. 1, the size- and shape-dependent melting temperatures *T*_m_(*D*,*λ*) of Au and Ni nanoparticles have been plotted in terms of Eq. (3) together with the necessary parameters listed in Tables 1 and 2. It is found that the melting temperature of nanoparticles decreases with decrease in the size or increase in the shape factor and the shape effect on *T*_m_(*D*,*λ*) becomes evident at small size. Available MD simulations results^34^,^35^,^36^,^37^,^38^,^39^ and experimental data^39^,^40^ are also listed for comparisons. Obviously, the model predictions agree with the corresponding experimental and MD simulations results, which indicates the validity and accuracy of Eq. (4) in determining the shape factor.

It is known that Ω = *ZN*_a_[*ε*_AB_ − (*ε*_AA_ + *ε*_BB_)/2] where *Z* denotes the coordinate number of an atom, *N*_a_ is the Avogadro’s constant, and *ε* denotes the bond energy^22^. The size dependence of interaction energy should also be the same as that of the cohesive energy, since both of them are proportional to the bond energy^20^,^21^,^22^. Combining Eq. (3) with the consideration of the composition effect, a unified model to describe size-, shape-, and composition-dependent interaction energy Ω(*x*,*D*,*λ*) can be deduced as^21^


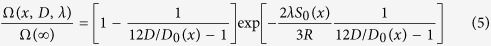


As a first-order approximation, the composition-dependent bulk vaporization entropy *S*_0_(*x*) and critical size *D*_0_(*x*) can be obtained by the Fox equation^42^,


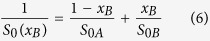



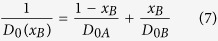


In terms of Eqs (1)–(7), size- and shape-dependent continuous binary nano-phase diagrams with and without surface segregation can be calculated. Ni-Cu and Cu-Au systems have been selected as typical examples due to their representative phase diagram shapes.

## Result & Discussion

**Figure 2:**
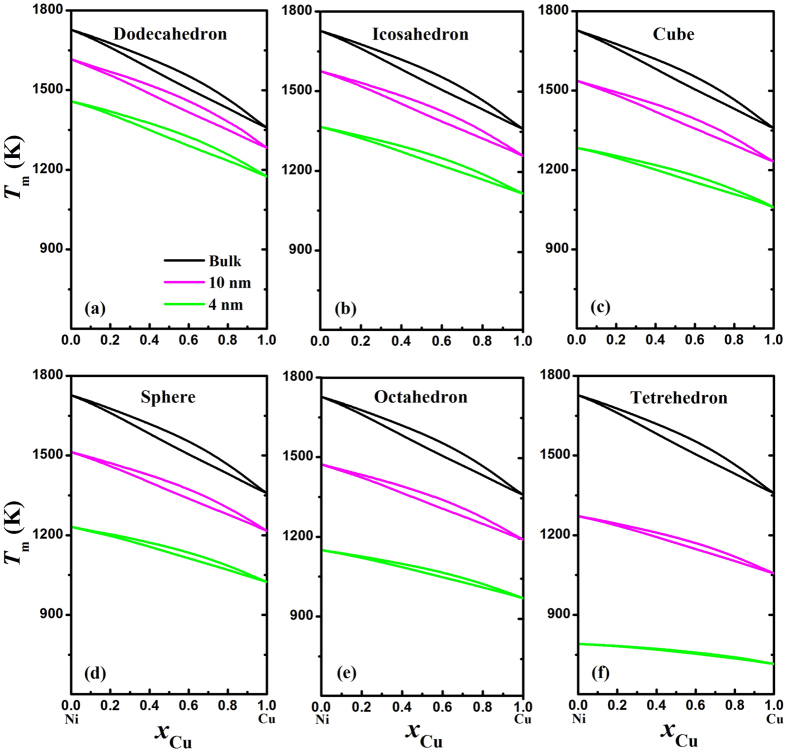
Phase diagrams of Ni-Cu polyhedrons at bulk, 10 nm and 4 nm for (**a**) dodecahedron, (**b**) icosahedron, (**c**) cube, (**d**) sphere, (**e**) octahedron, and (**f**) tetrahedron.

Figure 2 presents the predicted nano-phase diagrams of Ni-Cu alloy with different shapes in terms of Eqs (1) and (3)–(7), 11, 10, 11 at two sizes of 10 and 4 nm, where the bulk phase diagram is also calculated and listed for comparison. It can be found that both the solidus and the liquidus drop with decrease in the size and the two-phase zone diminish gradually because the difference between Ω_s_(*D*) and Ω_l_(*D*) decreases with the size. When the size decreases to a critical size D*, the regular solution deteriorates into the ideal solution and the two-phase zone vanishes where the liquid phase and the solid phase are indistinguishable in structure and thus the phase numbers transform from two to one, as shown in Fig. 2f. Note that *λ*_tetrahedron_ > *λ*_octahedron_ > *λ*_sphere_ > *λ*_cube_ > *λ*_icosahedron_ > *λ*_dodecahedron_ as listed in the Table 1, and thus the higher shape factor, the narrower two-phase zone and the larger critical size D*. Moreover, the melting temperature of nanoalloys also decreases with the increasing shape factor since the physical origin for the depression of melting temperature is thought of as the enormous ratio of the number of surface-to-volume atoms^43^, where the surface-to-volume ratio is related to the shape. By comparing the phase diagrams of different Ni-Cu polyhedral nanoalloys as shown in Fig. 2a–f, it can be found that the dodecahedron presents the higher solidus and liquidus than others at the same size, which implies that the most stable shape investigated theoretically of nanoalloys is dodecahedron. Nevertheless, the predictions can differ from the shapes observed experimentally due to the critical role played by defects and adsorbed species on the surface of nanoparticles^44^.

**Figure 3:**
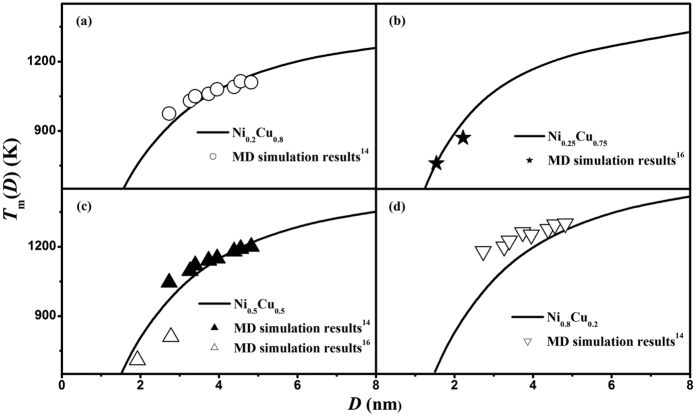
Comparisons of *T*_m_(*D*) functions for (**a**) Ni_0.8_Cu_0.2_, (**b**) Ni_0.75_Cu_0.25_, (**c**) Ni_0.5_Cu_0.5_, and (**d**) Ni_0.2_Cu_0.8_ nanoparticles between the model predictions and available MD simulation results^14^,^16^.

Because there are no systematic experimental investigations or computer simulations on the phase diagrams of binary nanoalloys over the whole composition range at the selected size (4 or 10 nm), no comparison between our model prediction and experimental data or computer simulation results are made in Fig. 2. However, some MD simulations have been carried out on the size-dependent melting temperature *T*_m_(*D*) of Ni-Cu nanoalloys at certain compositions, *i.e.* Ni_0.8_Cu_0.2_, Ni_0.75_Cu_0.25_, Ni_0.5_Cu_0.5_ and Ni_0.2_Cu_0.8_^14^,^16^. Figure 3 compares the *T*_m_(*D*) functions of these four Ni-Cu nanoalloys between our model predictions and the corresponding MD simulation results^14^,^16^, where good agreements can be found. Similar to the depression of *T*_m_(*D*) for pure elements as shown in Fig. 1, the melting temperature of nanoalloys also decreases with the decreasing size at a given composition and the drop becomes dramatic at *D* < 5 nm. Furthermore, as shown in Fig. 3, the melting temperature of Ni-Cu nanoalloys ascends with decrease in the composition of Cu at the same size.

**Figure 4:**
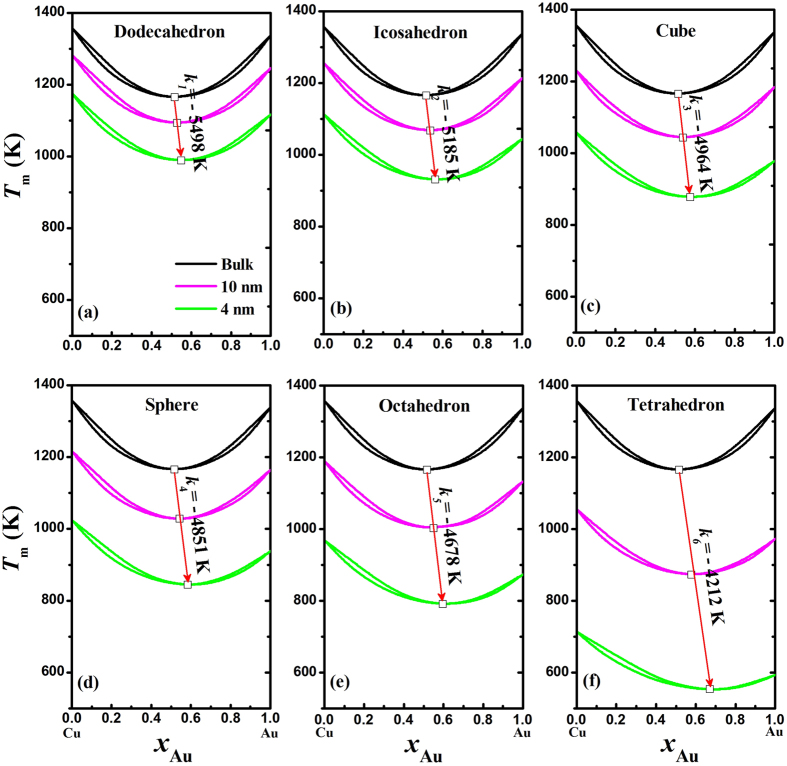
Phase diagrams of Cu-Au alloys at bulk, 10 nm, and 4 nm, where the symbol (□) denotes the congruent melting point and the arrow highlights the size and shape effects on the congruent melting point.

Different from the phase diagram of Ni-Cu where both the solidus and liquidus vary monotonically with increasing *x*_B_, there is an intersection between the solidus and liquidus curves in the phase diagram of Cu-Au. Note that the congruent melting point is lower than the melting temperature of either Cu or Au, implying the greater stability of the liquid solution than the solid one. Figure 4 presents the predicted nano-phase diagrams of Cu-Au alloy with different shapes in terms of Eqs (1) and (3)–(7), 11, 10, 11 at two sizes of 10 and 4 nm. The bulk phase diagram is also calculated and listed for comparison. It can be found that the reduction of two-phase zone becomes less distinguishable with the decreasing size than that in Ni-Cu nanoalloys, originating from the small difference of *S*_0_ between Cu and Au in comparison with that between Cu and Ni since *S*_0_ is the only variable parameter in Eqs (3) and (5)–(7, 10, 11) at certain composition, size and shape. The solidus and liquidus of bulk phase diagram of Cu-Au meet at the congruent melting point, which occurs at 52%Au composition and 1165 K. As shown in Fig. 4, the congruent melting point of the nano-phase diagrams is linearly shifted towards higher Au component and lower temperature with decreasing size. It is reasonable and understandable: Au possesses higher *S*_0_ value than Cu, and the size dependences of solidus and liquidus with higher Au component are stronger than those with lower Au component. Moreover, the higher shape factor, the larger slope of the arrow (*k*_1_ < *k*_2_ < *k*_3_ < *k*_4_ < *k*_5_ < *k*_6_), namely, the greater effect of shape factor on the shift of the congruent melting point.

Continuous binary nanoalloy with well-controlled size and shape is a remarkable catalyst, which has low catalytic activation energy and thus high rate of reaction^3^,^4^. Recently, Wang *et al*. have proposed that Au_0.5_Ni_0.5_ nanoalloys exhibited the lowest catalytic activation energy in comparison with Au_0.9_Ni_0.1_, Au_0.8_Ni_0.2_, Au_0.2_Ni_0.8_, and Au_0.1_Ni_0.9_ at similar size for a reaction of hydrogen generation^45^. It has been reported that size- and shape-dependent catalytic activation energy of nanocrystals is directly proportional to its melting temperature^19^,^32^. Since the shape of phase diagram of Au-Ni alloy is similar to that of Cu-Au alloy and the composition of Au_0.5_Ni_0.5_ is more closer to that of the congruent melting point (43%Ni composition) than other Au_1 − *x*_Ni_*x*_ alloys with *x* = 0.1, 0.2, 0.8 and 0.9, Au_0.5_Ni_0.5_ nanoalloy should have the lowest melting temperature and catalytic activation energy among these five Au-Ni nanoalloys, which agree with the experimental results of Wang^45^. This agreement also suggests that our model is valuable for the design of catalyst of nanoalloys.

The cohesive energy of solid is an important physical quantity to account for the binding strength of the crystal and estimate the stability of nanoalloys with different sizes and shapes. Similar to the pure elements, the cohesive energy of nanoalloys should also be directly proportional to its melting temperature when the structure remains the same^31^,


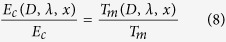


**Figure 5:**
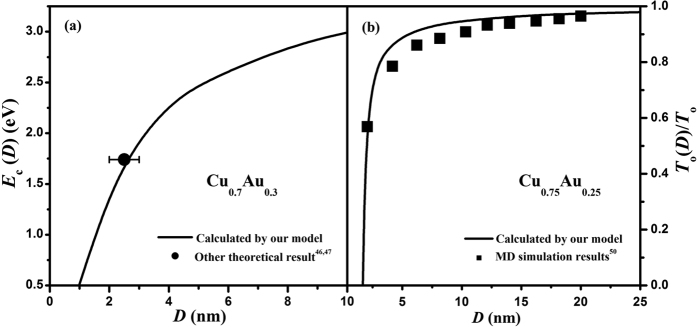
(**a**) *E*_c_(*D*) of Cu_0.7_Au_0.3_ nanoalloys as a function of *D* in terms of Eq. (8) where the bulk cohesive energy of Cu_0.7_Au_0.3_ is calculated by Fox equation and the symbol (●) is other theoretical result^46^,^47^. (**b**) *T*_o_(*D*)/*T*_o_ of Cu_0.75_Au_0.25_ nanoalloys as a function of *D* in terms of Eq. (9) where the symbol (■) denotes available MD simulation results^50^.

Harinipriya and Sangaranarayanan theoretically estimated the cohesive energy of disc-shaped Cu_0.7_Au_0.3_ with the wide D and height *L* being 2–3 and 1 nm to be about 1.74 eV^46^,^47^. Note that the disc-shaped nanocrystal can be assumed to have a quasi-dimensionality of *d* = 1^19^ and the shape factor of disc-shaped nanocrystal can be determined as *λ* = (*L* + *D*/2)/*L* in terms of Eq. (4). Since *T*_m_(*D*,*λ*,*x*) value of nanoalloys can be obtained through our established nano-phase diagram, *E*_c_(*D*,*λ*,*x*) of nanoalloys can be calculated in terms of Eq. (8). Figure 5a compares the *E*_c_(*D*) function of disc-shaped Cu_0.7_Au_0.3_ nanoalloys between the model prediction with *d* = 1 and *λ* = (*L* + *D*/2)/*L* ≈ 2.25 and other theoretical estimation^46^,^47^, where an agreement between model prediction and one data point can be found. The cohesive energy decreases with decrease in the size, which reflects the instability of nanoalloys in comparison with the corresponding bulk crystals. This trend is expected since the surface/volume ratio increases with decreasing size while the surface atoms have lower coordination number and higher energetic state, and consequently the low stability of nanoalloys^48^.

Not only the cohesive energy but also the order-disorder transition temperature *T*_o_(*D*) of nanoalloys can be determined through our established nano-phase diagram. It has been reported that the size dependence of *T*_o_(*D*) is directly proportional to the root-mean-square of that of *T*_m_(*D*)^49^, *i.e.*


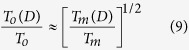


Figure 5b presents the normalized *T*_o_(*D*)/*T*_o_ function of Cu_0.75_Au_0.25_ spherical nanoparticles in terms of Eq. (9) with *λ* = 1, where *T*_o_(*D*) decreases with decreasing size and the drop becomes dramatic once the particle size decreases below 3 nm. Our model predictions are confirmed by MD simulation results^50^. According to Eq. (9), it is obvious that the size dependence of *T*_o_(*D*) is smaller than that of *T*_m_(*D*) at the same size.

**Figure 6:**
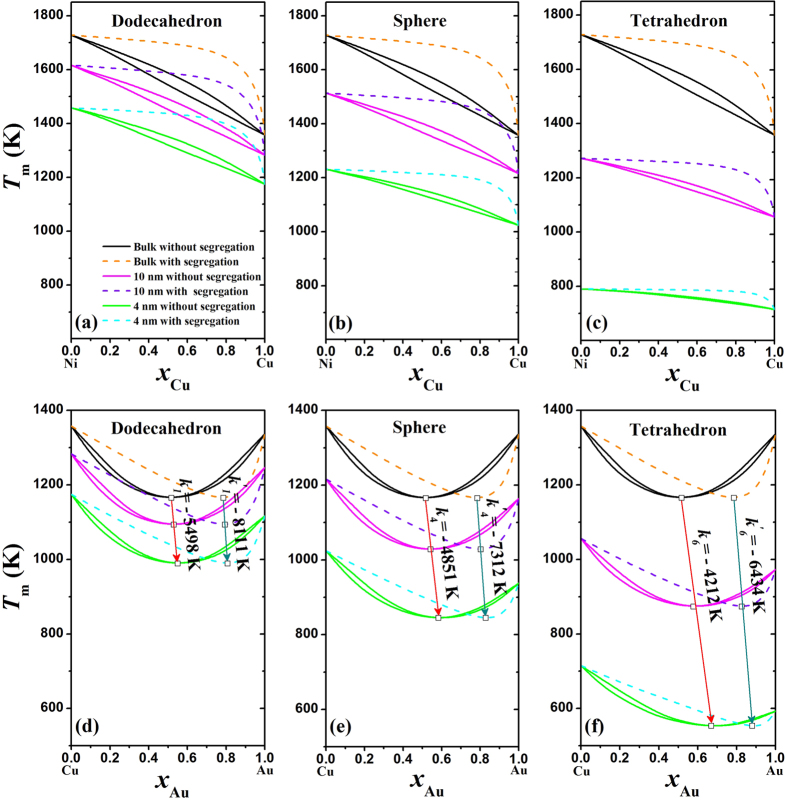
Solidus(core)/liquidus(core) and solidus(surface) of dodecahedral, spherical, and tetrahedral Ni-Cu (**a–c**) and Cu-Au (**d–f**) nanoalloys without and with surface segregation at bulk, 10, and 4 nm, where the red and cyan arrows are only to guide the eyes and highlight the size and shape effects on the congruent melting point, respectively.

As shown in Fig. 6, solidus(core)/liquidus(core) and solidus(surface) of Ni-Cu and Cu-Au nanoalloys with and without segregation are plotted for the most stable shape (dodecahedron), basal shape (sphere), and the least stable shape (tetrahedron). It is obvious that Cu and Au always segregate to the surfaces of Ni-Cu and Cu-Au nanoalloys, which agrees with the corresponding experimental results^28^,^29^. Red and cyan arrows in Fig. 6d–f are used to highlight the size and shape effects on the congruent melting point without and with segregation, respectively. It can be found that the congruent melting point in the nano-phase diagrams always shifts towards higher Au composition and lower temperature where the slope of cyan arrow is always smaller than that of red arrow at a given shape of nanoalloy (*k’* 1 < k_1_, *k’* 4* < k*_4_ and *k’* 6* < k*_6_), *i.e.* the occurrence of surface segregation causes the composition change to slow down at the congruent melting point. Moreover, the higher shape factor, the greater slope of the cyan arrow (*k’* 1* < k’* 4* < k’* 6), which indicates that the degree of segregation decreases with decrease in the size or increase in the shape factor. This changing trend agrees with the MD simulation results^29^. Based on elemental variables related to the work functions and electron densities of the elements, Hamilton concluded that the surface energy difference was the dominant driving force while the strain energy and the heat of solution played a minor role in determining segregation behaviour for most binary alloys^51^. Since the surface energy is also descended with decreasing size^27^, the drop in driving force for surface segregation is expected.

To predict the nature of the segregated element, preferentially found at the surface of the binary nanoalloys, we identified two segregation rules based on the solid surface energy γ_s_ and atomic size *h*: The first rule says that if the surface energy of element A is larger than the element B, then element B will segregate to the surface; When the surface energy difference between two elements is less than ~10% of the highest surface energy, then the element with the largest atomic size segregates to the surface to release the strain energy, this is the second rule. These two rules have been summarized in Table 3 to explain the surface segregation of different binary nanoalloys. For example, since γ_s_ of Ag is obviously smaller than those of Au, Co, Cu, Ni and Pd^27^, the segregated element in Au-Ag, Ag-Co, Ag-Cu, Ag-Ni and Ag-Pd alloys is Ag according to our first rule, in agreements with the corresponding experimental results^9^. Similar conditions occur in Au-Cu, Au-Ni, Au-Pd, Au-Pt, Cu-Ni, Pd-Ni and Pd-Pt alloys. While for Fe-Ni and Pt-Ni alloys with *h*(Fe) < *h*(Ni) < *h*(Pt)^52^, Ni and Pt respectively segregate to the surfaces according to our second rule, since the differences of γ_s_ among Fe, Ni and Pt are smaller than 5%^27^.

Remarkably, these two segregation rules are applicable when the binary alloys are not affected by external factors, such as heat treatment, adsorbents, substrates, and other inducements^30^,^53^, since these factors can drastically alter the surface energy of nanoalloys and thus directly modify the surface segregation. For example, Au with the lower surface energy segregates to the surface of Cu-Au nanoalloys. Nevertheless, the sample exposed to O_2_ would lead to a preference of Au to stay in the interior of Cu-Au nanoalloys, caused by the energetically much more favorable O binding to Cu than to Au atoms^29^.

## Concluding Remarks

A unified thermodynamic model based on the size-dependent cohesive energy model has been developed to predict the size, shape and segregation effects on phase diagrams of continuous binary nanoalloys. For Cu-Au nanoalloys with segregation, the congruent melting point is linearly shifted to higher Au composition and lower temperature and the degree of segregation decreases with decrease in the size or increase in the shape factor. Moreover, it is found that surface segregated elements are Cu and Au in Ni-Cu and Cu-Au nanoalloys for all the shapes investigated. Two segregation rules based on the solid surface energy and atomic size have been developed to predict the segregated elements when the binary alloys are not affected by external factors, and these rules agree with experimental measurements. Finally, the established nano-phase diagrams can be employed to describe the physicochemical of nanoalloys, and the validity is supported by available theoretical predictions, experimental data, and MD simulations results.

## Additional Information

**How to cite this article:** Cui, M. J. *et al*. Phase Diagram of Continuous Binary Nanoalloys: Size, Shape, and Segregation Effects. *Sci. Rep.* **7**, 41990; doi: 10.1038/srep41990 (2017).

**Publisher's note:** Springer Nature remains neutral with regard to jurisdictional claims in published maps and institutional affiliations.

## Figures and Tables

**Table 1:** Shape Parameters for Several Polyhedral Particles.

Shape of nanoparticles	*η*_S_	*A*_C_	*V*_C_	*λ*
Sphere with (111) facets	3^1/2^*π*/6	*πD*^2^	*πD*^3^/6	1
Tetrahedron with (111) facets	3^1/2^*π*/6	3^1/2^*D*^2^	2^1/2^*D*^3^/12	2.45
Cube with (100) facets	*π*/4	6*D*^2^	*D*^3^	0.87
Octahedron with (111) facets	3^1/2^*π*/6	12^1/2^*D*^2^	2^1/2^*D*^3^/3	1.22
Dodecahedron with (111) facets	3^1/2^*π*/6	15tan(54°)*D*^2^	(15 + 7 × 5^1/2^)*D*^3^/4	0.45
Icosahedron with (111) facets	3^1/2^*π*/6	5 × (3)^1/2^*D*^2^	(15 + 5 × 5^1/2^)*D*^3^/12	0.66

**Table 2:** Necessary Parameters Used to Calculate the Nano-Phase Diagrams.

	Au	Cu	Ni
*T*_m_ (K)^33^	1337	1358	1728
*H*_m_ (J/mol)^33^	12500	13100	17200
*h* (pm)^33^	288.4	255.6	249.2
*S*_0_ (J/mol·K)^33^	105.47	93.75	118.64
*Q*_seg_ (J/mol)^28^	11900		
		35500	
Ω^*s*^ (J/mol)^20^,^22^	−20290		
		11376	
Ω^*l*^ (J/mol)^20^,^22^	−27230		
		12219	

**Table 3:** Relationship between Solid Surface Energy and Atomic Diameter for Different Binary Alloys.

Alloy	γ_s_ (1^st^ rule)	*h* (2^nd^ rule)	Segregated element
Ag-Au	<		Ag^9^
Ag-Co	<		Ag^9^
Ag-Cu	<		Ag^9^
Ag-Ni	<		Ag^9^
Ag-Pd	<		Ag^9^
Au-Cu	<		Au^9^,^29^
Au-Ni	<		Au^9^
Au-Pd	<		Au^30^
Au-Pt	<		Au^9^
Cu-Ni	<		Cu^9^,^28^,^29^
Fe-Ni	~	<	Ni^9^
Pd-Ni	<		Pd^9^
Pt-Ni	~	>	Pt^9^
Pd-Pt	<		Pd^9^
